# Factors Associated With Gender Distribution in Academic Rhinology

**DOI:** 10.1002/oto2.70092

**Published:** 2025-02-26

**Authors:** Alison J. Yu, Stephanie Wong, Kevin Herrera, Daniel Palmieri, Bozena Wrobel, Tamara Chambers, Nadia Chan

**Affiliations:** ^1^ Department of Otorhinolaryngology University of Pennsylvania Philadelphia Pennsylvania USA; ^2^ Caruso Department of Otolaryngology–Head and Neck Surgery University of Southern California Los Angeles California USA

**Keywords:** academic otolaryngology, gender diversity, gender equality, female representation, rhinology workforce

## Abstract

**Objective:**

Women have been underrepresented in academic medicine and surgical fields. This study aimed to describe the gender distribution in academic rhinology and investigate otolaryngology departmental factors associated with female rhinologists representation.

**Study design:**

Cross‐sectional study.

**Setting:**

American Medical Association (AMA) FREIDA database, American Rhinologic Society database, and online search.

**Methods:**

We identified fellowship‐trained rhinologists from institutional searches of residency programs identified from the AMA FREIDA database. Demographic and academic data of each rhinologist were extracted from online search. Departmental characteristics were obtained from the FREIDA database and institutional websites. The associations between female rhinologists representation and departmental factors were assessed using logistic regression analyses.

**Results:**

Among 224 fellowship‐trained rhinologists, female comprised 26.8%. There were more female assistant professors (55.0% vs 39.6%) and fewer full professors (10.0% vs. 24.4%) than their male counterparts (*P* = .046). The mean *H* index was lower for female than male rhinologists among those over 5 years in practice (20.5 vs 33.0, *P* = .029). In the multivariate analysis, female rhinologists were more likely to be found in departments with greater proportions of female faculty (adjusted odds ratio 1.08 [95% confidence interval 1.04‐1.12], *P* < .001).

**Conclusion:**

The proportion of female rhinologists decreased significantly as they advanced through academic rank. The main driver for female rhinologists representation is the increased number of female faculty in the department. There should be increased efforts to promote female mentorship and women in leadership positions.

Prior studies have demonstrated that diversity in the healthcare workforce has been associated with positive impact on patient health outcomes and team environment.^1^, ^2^ There have been overall recent trends toward greater gender diversity in medical students and otolaryngology residents.^3^ In 2023, more than half of all medical students were female (55.4%).^4^ Despite greater diversity at the trainee level, women continue to be underrepresented in surgical specialties and in academic medicine.^5^, ^6^ Physicians in academic practice often face distinct challenges throughout their careers, such as academic promotion, diverse commitments at work, work life balance, and leadership responsibilities. Women make up 19% of academic otolaryngologists and approximately 37% of the physician workforce in academic medicine as of 2021.^7^, ^8^, ^9^ In the field of rhinology, a recent workforce analysis found that women comprised only 18% of all practicing rhinologists in the United States.^10^ However, by the 2023 US consensus bureau, women make up 51% of the general population.^11^ Given the unique obstacles faced by surgeons in academia, whether women are underrepresented in academic rhinology has not been investigated. It is also not clear what factors may be associated with a higher likelihood of having female rhinologists representation in the department.

In this study, we sought to understand the overall gender outlook in the field of academic rhinology. We also aimed to investigate various otolaryngology departmental factors that might influence the representation of female rhinologists.

## Materials and Methods

This was a cross‐sectional study conducted in September 2023 with data collected from several publicly available sources. This study was exempt from the University of Southern California Institutional Review Board approval as all data were publicly available. United States allopathic and osteopathic otolaryngology residency programs were obtained from the American Medical Association (AMA) FREIDA database. For each residency program, the institution's public website was searched to identify the rhinologist(s) in the department. Individuals were excluded if they were adjunct faculty or if they did not complete fellowship training in rhinology as stated on the institutional profile, American Rhinologic Society (ARS) member database, U.S. News and World Report, Doximity, or LinkedIn. Gender was determined by what was self‐reported on the institutional profile websites. Data regarding geographic practice location, academic degree(s), fellow of ARS status, and academic rank of the individual were extracted from the above sources. Years in practice was defined as years since the completion of fellowship with early career defined as 5 or fewer years, mid‐career 6 to 15 years, and late career more than 15 years. Individual H index was determined via Google Scholar author search.

Data regarding the departmental faculty size, residency size, proportion of female faculty, proportion of female residents, female chair or program director, number of rhinologists, and presence of a rhinology fellowship were obtained from the AMA FREIDA database and the institutional websites. Gender was determined by what was reported on these websites. Departmental National Institute of Health (NIH) funding level was obtained from the Blue Ridge Institute for Medical Research website based on the funding level in 2023 and was categorized by quartiles.

*χ* ^2^ or Fisher's exact test and independent *t* tests were used to compare individual and departmental data between female and male rhinologists. A power analysis was performed to ensure that the sample sizes were appropriate for the intended statistical analyses. Effect sizes were reported by Cramer's *V* estimate for comparing proportions and Cohen's *d* estimate for comparing mean differences for statistically significant results. Univariate and multivariable logistic regression analyses were used to determine the factors associated with having female rhinologist in the department. Statistical significance was defined as *P* < .05. All statistical analyses were performed using SPSS version 29 (IBM, New York).

## Results

In total, 224 fellowship‐trained rhinologists were identified. Female comprised 26.8% of the study population. There were more female assistant professors (55.0% vs 39.6%) and fewer female full professors (10.0% vs 24.4%) than their male counterparts (*P* = .046, Cramer's *V* estimate 0.19) (Table 1). More female rhinologists were in their early careers than their male colleagues although this was not statistically significant (40.7% vs 28.2%, *P* = .254, Cramer's *V* estimate 0.13). Time in practice by academic title was not significantly different between female and male rhinologists. Having multiple academic degrees was less prevalent among female than male rhinologists (11.7% vs 25.0%, *P* = .031, Cramer's *V* estimate 0.14). The mean *H* index was lower for female than male rhinologists among those who were over 5 years in practice (20.5 vs 33.0, *P* = .029, Cohen's *d* estimate 0.79) but this difference was not statistically significant for those who were over 15 years in practice (23.0 vs 43.1, *P* = .111).

**Table 1:** Demographics and Academic Information of the Study Population by Gender of the Rhinologist

	Total, N = 224	Male, N = 164 (73.2%)	Female, N = 60 (26.8%)	*P* value
Academic title, N (%)				.046*
Assistant professor	98 (43.8)	65 (39.6)	33 (55.0)	
Associate professor	71 (31.7)	51 (31.1)	20 (33.3)	
Professor	46 (20.5)	40 (24.4)	6 (10.0)	
N/A	9 (4.0)	8 (4.9)	1 (1.7)	
Years in practice, N (%)^a^				.254
≤5	70 (31.5)	46 (28.2)	24 (40.7)	
6‐15	115 (51.8)	87 (53.4)	28 (47.5)	
>15	37 (16.7)	30 (18.4)	7 (11.9)	
Geographic region, N (%)				.594
West	38 (17.0)	26 (15.9)	12 (20.0)	
Midwest	51 (22.8)	36 (22.0)	17 (28.3)	
South	67 (29.9)	50 (30.5)	17 (28.3)	
Northeast	64 (28.6)	50 (30.5)	14 (23.3)	
Puerto Rico	2 (0.9)	2 (1.2)	0	
Other degrees besides MD, N (%)	48 (21.4)	41 (25.0)	7 (11.7)	.031*
Fellow of ARS, N (%)	107 (47.8)	84 (51.2)	23 (38.3)	.087
Time in practice by academic title, years, mean (SD)				
Assistant professor	5.6 (4.7)	5.9 (5.0)	5.0 (4.1)	.363
Associate professor	10.5 (4.7)	10.5 (4.3)	10.6 (5.7)	.969
Professor	16.6 (5.1)	16.7 (5.3)	16.0 (3.8)	.743
N/A	6.8 (4.0)	7.4 (3.8)	2.0 (N/A)	.222
*H* index by time in practice, mean (SD)				
Over 5 years^b^	30.6 (16.5)	33.0 (16.6)	20.5 (11.9)	.029*
Over 15 years^c^	40.7 (16.6)	43.1 (15.3)	23.0 (21.2)	.111

Abbreviations: ARS, American Rhinologic Society; SD, standard deviation.

^a^ Data available for 222 rhinologists.

^b^ Data available for 51 rhinologists.

^c^ Data available for 17 rhinologists.

* *P* < 0.05.

For departmental factors, the mean proportion of female faculty was higher in the department with a female rhinologist than male (35.0% vs 26.8%, *P* < .001, Cohen's *d* estimate 0.80) (Table 2). A higher proportion of female rhinologists worked in a department with a female chair or program director than male rhinologists, although not statistically significant (56.7% vs 42.1%, *P* = .052). The number of faculty, residents, and rhinologists were not significantly different between the departments of the female and male rhinologists.

**Table 2:** Characteristics of the otolaryngology department by gender of the rhinologist

	Total, N = 224	Male, N = 164 (73.2%)	Female, N = 60 (26.8%)	*P* value
Number of faculty, mean (SD)	29.6 (23.8)	30.4 (25.0)	27.4 (20.3)	.404
Number of residents, mean (SD)	17.4 (6.7)	17.2 (6.6)	17.8 (6.8)	.560
Number of rhinologists, mean (SD)	3.1 (1.6)	3.2 (1.6)	3.0 (1.6)	.514
Proportion of female faculty, mean (SD)	29.0 (11.0)	26.8 (10.1)	35.0 (11.0)	<.001*
Proportion of female residents, mean (SD)	43.3 (13.3)	43.4 (13.7)	42.8 (12.4)	.759
Rhinology fellowship, N (%)	83 (37.1)	66 (40.2)	17 (28.3)	.102
Female chair/PD, N (%)	103 (46.0)	69 (42.1)	34 (56.7)	.052
Department NIH Funding, N (%)				.841
1st quartile	35 (15.6)	26 (15.9)	9 (15.0)	
2nd quartile	27 (21.1)	19 (11.6)	8 (13.3)	
3rd quartile	23 (10.3)	19 (11.6)	4 (6.7)	
4th quartile	24 (10.7)	18 (11.0)	6 (10.0)	
Unranked	115 (51.3)	82 (50.0)	33 (55.0)	

Abbreviations: PD, program director; SD, standard deviation.

* *P* < .05.

In the univariate logistic regression analyses, female rhinologists were more likely to be found in a department with a greater proportion of female faculty (odds ratio [OR] 1.08 [95% confidence interval [CI] 1.05‐1.12], *P* < .001) (Table 3). In the multivariate analysis, after controlling for departmental factors, greater proportion of female faculty remained significantly associated with female rhinologists representation (adjusted OR 1.08 [95% CI 1.04‐1.12], *P* < .001). There was no significant difference in the number of faculty (*P* = .816), number of residents (p = 0.490), number of rhinologists (*P* = .996), proportion of female residents (*P* = .180), or NIH funding level (*P* = .394‐.983) between the departments with female and male rhinologists.

**Table 3:** Factors Associated With the Representation of Female Rhinologists in the Department

	Univariate analysis		Multivariable analysis	
	Odds ratio (CI)	P value	Adjusted OR (CI)	*P* value
Number of faculty	0.99 [0.98‐1.01]	0.406	1.00 [0.98‐1.02]	.816
Number of residents	1.01 [0.97‐1.06]	0.558	1.03 [0.95‐1.12]	.490
Number of rhinologists	0.94 [0.78‐1.14]	0.512	1.00 [0.75‐1.33]	.996
Proportion of female faculty	1.08 [1.05‐1.12]	<0.001*	1.08 [1.04‐1.12]	<.001*
Proportion of female residents	1.00 [0.97‐1.02]	0.758	0.98 [0.95‐1.01]	.180
Rhinology fellowship	0.59 [0.31‐1.12]	0.104	0.42 [0.17‐1.01]	.053
Female chair/PD	1.80 [0.99‐3.27]	0.054	1.53 [0.75‐3.11]	.150
Department NIH Funding				
1st quartile	Reference		Reference	
2nd quartile	0.82 [0.54‐0.98]	0.732	1.01 [0.30‐3.47]	.983
3rd quartile	1.64 [1.24‐2.05]	0.460	0.53 [0.12‐2.28]	.394
4th quartile	1.04 [0.76‐2.87]	0.951	0.54 [0.12‐2.38]	.414
Unranked	0.86 [0.45‐2.72]	0.731	0.82 [0.25‐2.69]	.748

Abbreviations: CI, confidence interval; OR, odds ratio; PD, program director.

* *P* < .05.

## Discussions

In the present study, we found that women comprised 26.8% of academic rhinologists in the United States with more assistant professors and fewer full professors than their male counterparts. The representation of female rhinologists was associated with a greater percentage of female faculty in the department.

The proportion of female rhinologists in our study is comparable to those seen in other subspecialties in otolaryngology. A recent report of academic facial plastics surgeons demonstrated that 25.6% were female and of academic neurotologists 13.6%.^12^, ^13^ The finding of more female rhinologists in the assistant professorship position compared to their male colleagues could indicate a recent influx of earlier career female rhinologists into academic positions. A contemporary analysis of ARS fellowship data showed that 19.6% of the rhinology fellows have been female with an increase in the number of female fellows from 18.8% to 20.8% over the past two decades, suggesting recent modest improvement in gender parity in the pipeline.^14^ This trend mirrors the increasing proportion of female otolaryngology residents, which saw a 8% increase from 2008 (30%) to 2020 (38%).^3^, ^15^ Although female representation in otolaryngology has been improving, it still lags behind other surgical specialties such as neurosurgery and thoracic surgery.^3^ It is our hope that greater gender diversity among recent trainees will translate to increased female representation in academic rhinology in the coming years.

However, the proportion of female rhinologists dropped off significantly as they advanced through academic ranks as demonstrated by fewer female rhinologists who were full professors compared to their male colleagues. This “leaky pipeline” phenomenon, which has been used to describe less attrition as one advances through the academic rank, has been mentioned in numerous previous studies for other fields in medicine.^16^, ^17^, ^18^ It has been observed that female otolaryngologists tend to become more productive later in their career, suggesting a career lag compared to their male colleagues.^19^ This is also supported by our data where female rhinologists in their early careers were significantly less productive than their male counterparts with this difference becoming insignificant when comparing those in late careers. This career lag could be detrimental to female rhinologists as early productivity is important for academic advancement, with a recent study showing that the lower number of publications among early career female faculty contributed to the gender gap in promotion.^20^ The reason for this lag is likely multifactorial and can be related to the childbearing years overlapping with early career years, increased family and childcare responsibilities, and the lack of mentorship during the early career stage.

We found that the main driver for the representation of female rhinologists was the increased number of female faculty and leaders in the department. This positive association is consistent with a similar correlation seen between greater percentage of female otolaryngology residents and female faculty representation.^21^, ^22^ A survey study found that 35% of the female medical students had been discouraged from surgical careers by the lack of female role models.^23^ This concept of gender‐congruent role models has also been shown by another survey study that found that female otolaryngology applicants were more likely than male applicants to be influenced by the presence of female residents and faculty in the department when ranking programs.^22^ A similar relationship between residents and faculty from underrepresented minority groups has been reported.^24^ Females within medicine may feel a stronger sense of career compatibility when they have other females within their field. This trickle‐down effect is not only present from faculty to residents but is also pervasive among female faculty themselves. Similar to our findings, a previous study noted that female otolaryngology faculty were more likely to work in a department with a female chair.^21^ A potential explanation is that a more diverse faculty cohort may lead to the recruitment of diverse applicants as it can increase the overall attractiveness of the department. Female faculty may also choose to return to work at their training institutions that already have increased number of female residents. It is important to understand to what extent this trickle‐down effect may impact the distribution of female faculty in rhinology.

There is a growing body of literature discussing the importance of female mentorship in surgical specialties. Women who had female mentors were found to have higher number of publications and involvement in research in addition to more career satisfaction.^25^ For this purpose, the Women in Rhinology (WIR) section of the ARS was established in 2018 to serve as an arena to foster networking and mentorship opportunities between female rhinologists. Indeed, a recent survey of the ARS community revealed that more women than men agreed that mentorship during residency affected their decision to become a rhinologist.^14^ Numerous proposals have been suggested to further bolster female mentorship, including designating peer support and mentorship programs for females at the faculty and resident levels especially early on in their careers in order to ameliorate the leaky pipeline phenomenon. Early exposure to the field of rhinology among female medical students may also be beneficial.

Efforts to address inequalities in gender representation at the leadership level will likely have an impact on the distribution of female faculty in the department. However, women are still underrepresented in leadership positions in otolaryngology. In 2022, 11.8% of the otolaryngology department chairs were female.^26^ Female editorial board membership in major otolaryngology journals stood at 17.7% in 2017.^27^ Specifically in rhinology, 5.5% of the ARS fellowship directors were female.^14^ Interventions to help increase female leadership have included emphasizing faculty diversity in department reviews by chairs and reducing selection bias during faculty selection with the blinding of applications from name and gender. It remains to be seen whether these interventions will lead to improved gender parity at the leadership level.

There are several limitations to this study. Due to the cross‐sectional nature of the study, the causality relationship between the distribution of female rhinologists and increased number of female faculty in the department cannot be determined. There are multiple factors that may come into play during the decision of choosing an institution to work at that are outside of the scope of our study. It would be interesting to have longitudinal data to ascertain the number of years that the individuals have spent in a certain academic rank to elucidate the timing of promotion as well as to assess changes in the workforce over time. Other demographics information of the study cohort including race/ethnicity and age were not available in this dataset. The accuracy of our data may be limited by the fact that the information was compiled from publicly available sources. Finally, the study population was restricted to fellowship trained rhinologists in academic institutions so our conclusions may not be applicable to rhinologists outside of this setting. Future studies including additional demographic factors and incorporating the attitudes and opinions of current academic rhinologists may assist in identifying the barriers to diversity in the workplace and ways to address them.

## Author Contributions

**Alison J. Yu**, design, conduct, analysis, presentation; **Stephanie Wong**, design, conduct, presentation; **Kevin Herrera**, design, conduct, presentation; **Daniel Palmieri**, design, conduct, presentation; **Bozena Wrobel**, design, conduct, presentation; **Tamara Chambers**, design, conduct, analysis, presentation; **Nadia Chan**, design, conduct, presentation.

## Disclosures

### Competing interests

The authors declare no conflicts of interest.

### Funding source

None.
